# Contact patterns of UK home delivery drivers and their use of protective measures during the COVID-19 pandemic: a cross-sectional study

**DOI:** 10.1136/oemed-2022-108646

**Published:** 2023-04-13

**Authors:** Jessica R E Bridgen, Hua Wei, Carl Whitfield, Yang Han, Ian Hall, Chris P Jewell, Martie J A van Tongeren, Jonathan M Read

**Affiliations:** 1 Centre for Health Informatics, Computing and Statistics, Lancaster Medical School, Lancaster University, Lancaster, UK; 2 School of Health Sciences, University of Manchester, Manchester, UK; 3 Immunity and Respiratory Medicine, University of Manchester, Manchester, UK; 4 Department of Mathematics, University of Manchester, Manchester, UK; 5 Department of Mathematics and Statistics, Lancaster University, Lancaster, UK; 6 Academic Health Sciences Centre, University of Manchester, Manchester, UK

**Keywords:** COVID-19, epidemiology, occupational health, viruses, disease outbreaks

## Abstract

**Objectives:**

To quantify contact patterns of UK home delivery drivers and identify protective measures adopted during the pandemic.

**Methods:**

We conducted a cross-sectional online survey to measure the interactions of 170 UK delivery drivers during a working shift between 7 December 2020 and 31 March 2021.

**Results:**

Delivery drivers had a mean number of 71.6 (95% CI 61.0 to 84.1) customer contacts per shift and 15.0 (95% CI 11.2 to 19.2) depot contacts per shift. Maintaining physical distancing with customers was more common than at delivery depots. Prolonged contact (more than 5 min) with customers was reported by 5.4% of drivers on their last shift. We found 3.0% of drivers had tested positive for SARS-CoV-2 since the start of the pandemic and 16.8% of drivers had self-isolated due to a suspected or confirmed case of COVID-19. In addition, 5.3% (95% CI 2.3% to 10.2%) of participants reported having worked while ill with COVID-19 symptoms, or with a member of their household having a suspected or confirmed case of COVID-19.

**Conclusion:**

Delivery drivers had a large number of face-to-face customer and depot contacts per shift compared with other working adults during this time. However, transmission risk may be curtailed as contact with customers was of short duration. Most drivers were unable to maintain physical distance with customers and at depots at all times. Usage of protective items such as face masks and hand sanitiser was widespread.

**WHAT IS ALREADY KNOWN ON THIS TOPIC**

- Contact patterns are thought to drive occupational variation in risk of SARS-CoV-2 infection and workplace outbreaks.
- The home delivery sector performed an important service to clinically vulnerable individuals shielding at home, however, the type and number of interactions made by delivery drivers has not been quantified.

**WHAT THIS STUDY ADDS**

- Delivery drivers made a larger than average number of contacts per shift, when compared with the general workforce at this time, though contact with customers was of short duration.
- Some drivers reported working while they or a household member were ill with or suspected of having COVID-19 for financial reasons.

**HOW THIS STUDY MIGHT AFFECT RESEARCH, PRACTICE OR POLICY**

- Paid sick leave for delivery drivers may help to improve adherence to self-isolation and reduce SARS-CoV-2 infection risk to the workforce and customers.

## Introduction

The delivery sector has been central in ensuring that services and supplies have remained available throughout the COVID-19 pandemic in the UK. There has been an unprecedented demand for home deliveries during the pandemic, rising sharply with the implementation of nationwide ‘stay at home’ orders.^1^ The UK government classed delivery drivers as key workers, defined as workers critical to the COVID-19 response.^2^ Shielding guidance for clinically extremely vulnerable individuals in January 2021 advised the use of food and prescription delivery services to minimise the need to leave home.^3^ As non-essential businesses were forced to close for extended periods of time in 2020 and 2021, many businesses relied on online sales to generate income.^4^

Transmission of SARS-CoV-2, the virus that causes COVID-19, primarily occurs through airborne routes, however, indirect transmission may occur through contaminated surfaces.^5 6^ High contact occupations are thought to be associated with an increased likelihood of employees being exposed to SARS-CoV-2 and developing clusters of cases in the workplace.^7 8^ Reducing the number of social contacts, increasing ventilation and frequent handwashing were advised methods to reduce workplace risk of exposure to SARS-CoV-2.^9^ Delivery drivers interact regularly with other employees at depots (or collection hubs) and with a large number of customers. To mitigate against infection, contact-free deliveries became widely available to minimise contact and reduce transmission risk between delivery drivers and customers.^10^

The study period, 7 December 2020 to 31 March 2021, coincides with the peak and gradual decline of the second wave of COVID-19 in the UK, following the emergence of the alpha variant.^11 12^ The UK also entered a period of lockdown during this time, where ‘stay at home’ orders were in place, and non-essential businesses were closed to reduce transmission.^13^ We aimed to quantify the contact patterns of delivery drivers within their depot and with their customers, identifying the types of contact they were making.

## Methods

### Survey methodology

We conducted a cross-sectional online survey between 7 December 2020 and 31 March 2021 to quantify behaviours thought to be associated with transmission risk of SARS-CoV-2. An anonymous online survey (the ‘CoCoNet: Home Delivery Driver survey’) was used for data collection. The survey design was adapted from a previous population-wide study.^14^ Study participants had to meet the following inclusion criteria: a resident in the UK at the time of the survey, working as a home delivery driver and aged 18 or over. The survey was promoted through university press releases, engagement with delivery driver groups on social media (LinkedIn and Facebook) and targeted Facebook advertisements.

Survey responses received between 7 December 2020 and 31 March 2021 were included in the analysis. Partial survey responses were analysed for all questions that had been displayed to the participant.

Age, sex, ethnicity, household size and other demographic information was collected from participants. Employment information was requested, including employment type, working hours, types of items typically delivered and sick pay eligibility. The survey included questions pertaining directly to COVID-19, such as whether participants had tested positive for SARS-CoV-2, if they had to self-isolate due to suspected or confirmed SARS-CoV-2 infection and if they had worked while being ill with COVID-19 symptoms. Participants were asked to recall specific details from their last shift working as a delivery driver, including the number of customers they met face to face, the number of individuals they had a face-to-face conversation with at their depot and their use of personal protective equipment (PPE). The survey questions can be found in online supplemental material and the dataset is publicly available.^15^

10.1136/oemed-2022-108646.supp1 Supplementary data

### Primary and secondary outcome measurements

Our primary outcome measurement was the number of contacts delivery drivers have per shift. This was stratified into contact with customers and contact with individuals (employees or customers) at a delivery depot. A contact was defined as someone whom the participant had a face-to-face conversation with. Secondary outcome measurements were: the number of deliveries per shift, the type of contact drivers were having with customers, ability to maintain physical distance, use of protective items, COVID-19-related presenteeism, the frequency of self-isolation and COVID-19 infection.

### Data analysis

Study representativeness was assessed by comparing participant demographics with the labour force survey (LFS) estimates for delivery drivers and couriers. Quarterly LFSs for the time period November 2020 to January 2022 were aggregated for comparison, due to the relatively small sample size of delivery drivers and couriers included in each individual survey.^16–21^ To assess representativeness, we compared the percentage distribution of each demographic variable from the LFS to the binomial CIs of our sample.

To identify occupational and personal characteristics associated with participants’ interactions with customers, we fitted a negative binomial regression model to the number of customer contacts per shift. Explanatory variables included in the model were: age, sex, employment type, furthest distance travelled from the collection point to a delivery, weekly working hours and the type of items delivered. The model was assessed for multicollinearity by calculating the variance inflation factor for each independent variable. Statistical analyses were conducting by using R V.4.0.2.

## Results

### Participant demographics

We received 170 survey responses between 9 December 2020 and 31 March 2021, which met our inclusion criteria. Male participants accounted for 75.3% (128/170) of the sample, our survey over sampled females when compared with the aggregated LFS (table 1). The majority of participants were aged 40–59 (56.5%, 96/170). Participants predominantly resided in England (81.8%, 139/170), with 1.8% (3/170) of participants residing in Northern Ireland, 10.6% (18/170) residing in Scotland and 5.9% (10/170) residing in Wales. Our sample was representative by nation when compared with the LFS. The distribution of ethnicities in our sample is broadly reflective of the sector when compared with the LFS aggregated data. Most participants in our sample were white (91.8%, 156/170) which is comparable to the LFS (92.3%, 1648/1785), however we also have representation of other ethnicities (table 1).

**Table 1:** Participant demographics and aggregated labour force survey estimates for ‘delivery drivers and couriers’

	No of participants (percentage, 95% binomial CI)	Aggregated Quarterly labour force survey estimates for delivery drivers and couriers*^16–21^ November 2020–January 2022 No of participants (percentage)
Age group	N=170†	N=1785
<18	–‡	5 (0.3%)
18–29	27 (15.9%, 10.74% to 22.26%)	230 (12.9%)
30–39	35 (20.6%,14.78% to 27.45%)	245 (13.7%)
40–49	46 (27.1%, 20.54% to 34.39%)	338 (18.9%)
50–59	50 (29.4%, 22.68% to 36.87%)	530 (29.7%)
60–69	12 (7.1%, 3.70% to 12.01%)	384 (21.5%)
70+	0 (0.0%, 0.00% to 2.15%)	53 (3.0%)
Sex	N=170†	N=1785
Female	40 (23.5%, 17.37% to 30.63%)	181 (10.1%)
Male	128 (75.3%, 68.11% to 81.58%)	1604 (89.9%)
Prefer not to say	2 (1.2%, 0.14% to 4.19%)	–
Ethnicity	N=170	N=1785
White	156 (91.8%, 86.57% to 95.42%)	1648 (92.3%)
Mixed/multiple ethnic groups	5 (2.9%, 0.96% to 6.73%)	9 (0.5%)
Asian/Asian British	5 (2.9%, 0.96% to 6.73%)	88 (4.9%)
Black/African/Caribbean/Black British	1 (0.6%, 0.01% to 3.23%)	26 (1.5%)
Other ethnic groups	0 (0.0%, 0.00% to 2.15%)	14 (0.8%)
Prefer not to say	3 (1.8%, 0.37% to 5.07%)	–
No response	0.00 (0%, 0.00% to 2.15%)	0 (0.0%)
Nation	N=170†	N=1785
England	139 (81.8%, 75.13% to 87.26%)	1491 (83.5%)
Northern Ireland	3 (1.8%, 0.37% to 5.07%)	79 (4.4%)
Scotland	18 (10.6%, 6.40% to 16.22%)	114 (6.4%)
Wales	10 (5.9%, 2.86% to 10.55%)	101 (5.7%)

N is the number of participants who provided a response to the question.

*Main occupation of participant recorded as ‘delivery drivers and couriers’.

†Question required a response from the participant.

‡Age group did not meet study inclusion criteria.

### Employment situation

The majority of participants reported their employment situation to be either self-employed and completely independent (54.3%, 95% CI 46.3% to 62.2%) or employed full time by one company (28.4%, 95% CI 21.6% to 36.0%) (see online supplemental table S1). Over half of delivery drivers (53.1%, 95% CI 45.1% to 61.0%) reported their weekly working hours to be between 31 and 50 hours, and 22.2% (95% CI 16.1% to 29.4%) of delivery drivers reported working more than 50 hours a week. The majority of participants reported their most recent working shift to be during the week of completing the survey (92.6%, 95% CI 87.4% to 96.1%). A small proportion of participants reported their most recent working shift to be more than a month before completing the survey (2.5%, 95% CI 0.7% to 6.2%). Most participants (68.2%, 95% CI 60.1% to 75.5%) reported that they did not receive statutory sick leave pay while working as delivery drivers.

### Workplace interactions

The mean number of customer contacts was 71.6 (95% CI 61.0 to 84.1) per shift. We found 95.2% (95% CI 90.4% to 98.1%) of participants had brief face-to-face interactions (less than 5 min) with customers on their last shift, 5.4% (95% CI 2.4% to 10.4%) of participants had prolonged face-to-face interactions (more than 5 min) with customers and 8.2% (95% CI 4.3% to 13.8%) had entered a customer’s property. We found that 61.9% (95% CI 53.5% to 69.8%) of participants were able to maintain physical distance with customers at all times during their last shift. A small proportion of participants (2.7%, 95% CI 0.7% to 6.8%) reported that they could not maintain physical distance at all on their last shift.

The mean number of contacts per shift in depot (where drivers collected items for delivery) was 27.9 (95% CI 12.2 to 55.8). This was reduced to 15.0 (95% CI 11.2 to 19.2) contacts per shift after excluding a single individual who reported making an exceptionally large number of contacts. We found that 42.4% (95% CI 34.2% to 50.9%) of participants reported that they were able to maintain physical distance from contacts at the depot at all times during their last shift. Whereas 8.3% (95% CI 4.4% to 14.1%) of participants were unable to maintain physical distance at all on their previous shift.

We found 10.5% (95% CI 6.1% to 16.4%) of participants shared a vehicle with a colleague during their last working week, of which, 56.2% (95% CI 29.9% to 80.2%) reported sharing the vehicle with the same colleague throughout the week.

**Figure 1:**
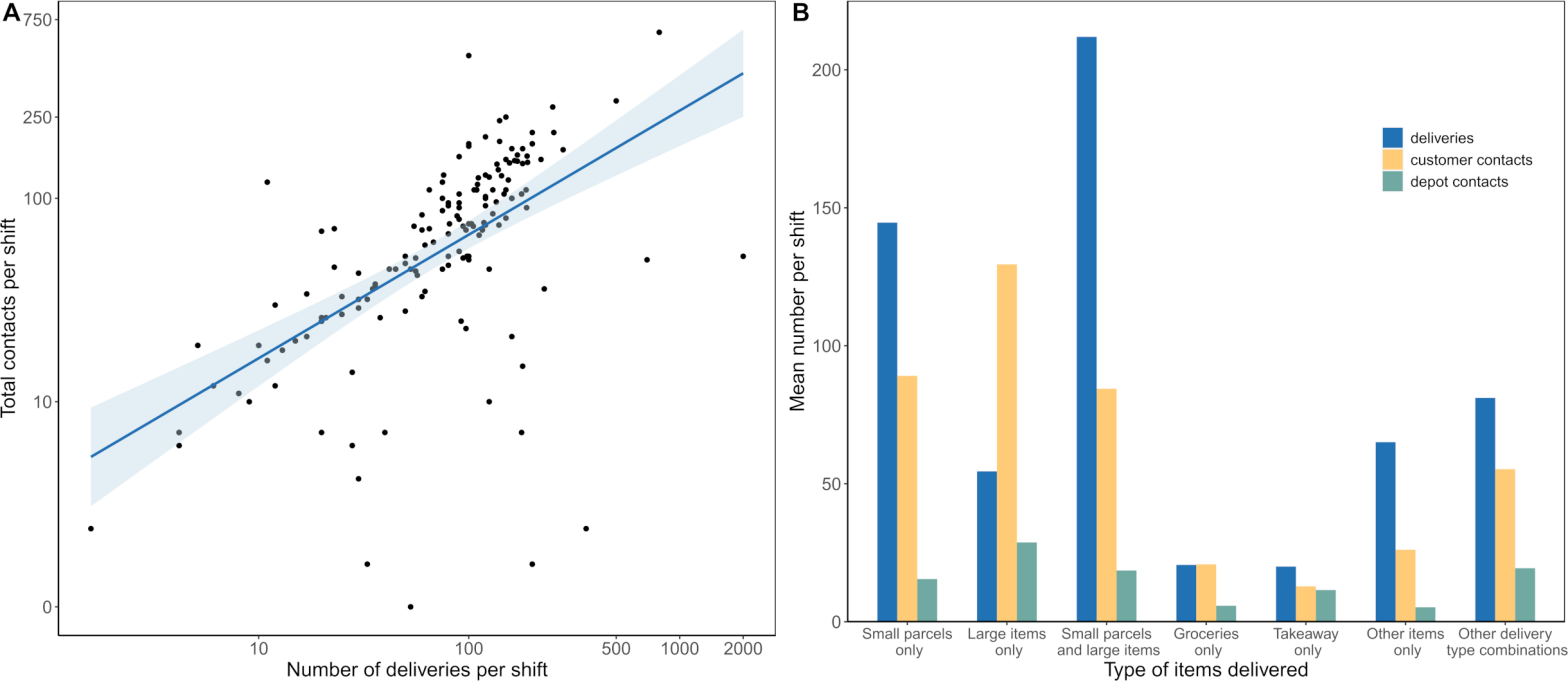
(A) Number of total contacts and deliveries made per shift. Note, x-axis and y-axis on log_10_-scale. Line and shaded area are a linear regression model. (B) Mean number of deliveries and contacts per shift by delivery type.

The number of contacts made per shift, including both customer and depot interactions, was positively correlated with the number of deliveries made per shift (figure 1A). Participants who reported typically delivering only large items (eg, large appliances, furniture) had the greatest number of customer and depot contacts per shift, making on average more customer contacts than deliveries per shift (figure 1B). While most drivers delivering large items only reported a one-to-one ratio of customer contacts and deliveries, one individual reported four times the number of customer contacts than deliveries.

### Frequency and type of deliveries

We found that the mean number of deliveries per shift was 121.8 (95% CI 97.9 to 152.3). Approximately half of participants (51.0%, 95% CI 42.8% to 59.1%) reported that the furthest distance they travelled from a collection point to a delivery address during their last working week was under 20 miles. The majority of delivery drivers surveyed (52.5%, 95% CI 44.5% to 60.4%) reported that they typically delivered small parcels (including letters and mail). We found that drivers delivering small parcels and large items had the highest mean number of deliveries per shift, while takeaway and grocery delivery drivers had the lowest (figure 1B).

### Predictors of customer contacts

**Figure 2:**
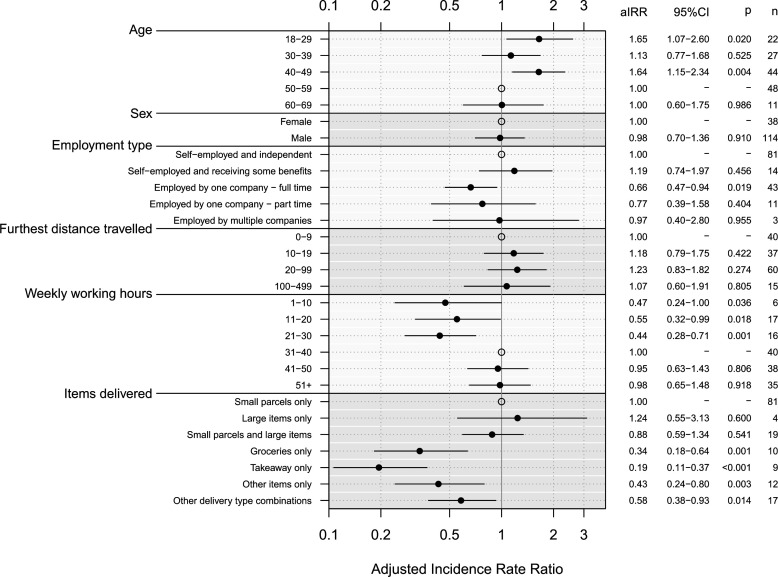
Adjusted incidence rate ratios (aIRR) for mean number of customer contacts reported for selected variables. Explanatory variables included in the model: age, sex, employment type, furthest distance travelled from the collection point to a delivery, weekly working hours and the type of items delivered. Open circles represent the reference group for each variable.

A negative binomial model was fitted to the number of face-to-face customer interactions per shift. The variance inflation factor was less than five for all model coefficients indicating multicollinearity was not present. Participants aged 18–29 (adjusted incidence rate ratio (aIRR) 1.65, 95% CI 1.07 to 2.60) and aged 40–49 (aIRR 1.64, 95% CI 1.15 to 2.34) had a higher number of customer contacts per shift than those aged 50–59 (figure 2, online supplemental table S2). We found that delivery drivers who were employed by one company full time had a lower number of customer contacts per shift than those self-employed and independent (aIRR 0.66, 95% CI 0.47 to 0.94). Participants who usually deliver only groceries (aIRR 0.34, 95% CI 0.18 to 0.64), deliver only takeaways (aIRR 0.19, 95% CI 0.11 to 0.37), deliver other unlisted items (aIRR 0.43, 95% CI 0.24 to 0.80) and those who deliver other combinations of items listed (aIRR 0.58, 95% CI 0.38 to 0.93) had fewer customer contacts than those that usually delivery only small parcels.

### COVID-19 infection, self-isolation and presenteeism

We asked respondents about their COVID-19 infection status to date, as well as their behaviour following infection or potential exposure; we examined these aspects independently. We found that 3.0% (95% CI 1.0% to 6.9%) of delivery drivers surveyed reported that they had tested positive for COVID-19 since the start of the pandemic. In addition, 16.8% (95% CI 11.4% to 23.3%) of delivery drivers had self-isolated since the start of the pandemic due to a suspected or confirmed case of COVID-19. Approximately 1 in 20 drivers (5.3%, 95% CI 2.3% to 10.2%) reported that they have continued to work while either being ill with COVID-19 symptoms or with a member of their household having a suspected or confirmed case of COVID-19. In this situation, financial reasons were most often cited as a reason for continuing to work (85.7%, 95% CI 42.1% to 99.6%).

### Protective measures

We found that 68.3% (95% CI 60.0% to 75.7%) of participants were provided with some PPE items by their employers or contracting companies. However, less than half of participants (48.3%, 95% CI 39.9% to 56.7%) felt that the PPE provided effective protection. Face masks (82.4%, 95% CI 75.4% to 88.0%) and hand sanitiser (83.7%, 95% CI 76.8% to 89.1%) were the protective items most commonly used by delivery drivers on their last shift. Participants who shared a vehicle during their last working week most often reported using hand sanitiser to prevent infection when sharing a vehicle (81.2%, 95% CI 54.4% to 96.0%), with 50.0% (95% CI 24.7% to 75.3%) of participants reporting wearing a face mask and 50.0% (95% CI 24.7% to 75.3%) reporting keeping a window open.

## Discussion

We found that delivery drivers made a large number of contacts per shift both at their depot (15.0 per shift) and with customers (71.6 per shift). In comparison, Jarvis and Edmunds found that the mean number of contacts among the general population attending their workplace between January 2021 and March 2021 was between 3 and 10 contacts per day; this included contacts made outside of the workplace.^22^ This suggests delivery drivers have a very large number of contacts compared with the general workforce at this time, which may lead to a higher risk of SARS-CoV-2 infection. The importance of contact duration in respiratory virus transmission has been widely documented.^23–27^ Face-to-face interactions between delivery drivers and customers are likely to take place outside and to be very short in duration, with only 5.4% of drivers reporting any prolonged contact (more than 5 min) with customers during their last working shift. Therefore, while delivery drivers have a large number of contacts, this may pose only a small risk in terms of exposure and transmission of SARS-CoV-2. Similarly, sharing a vehicle with a colleague for deliveries may be a type of high-risk contact. Nevertheless, as we found most work-related vehicle sharing was fixed-pairing (pair that share a vehicle is fixed) the risk of multiple transmission events is likely to be largely reduced. The duration of contacts made at the depot was not recorded.

The use of protective measures in the workplace was common. Most delivery drivers reported being able to maintain physical distance with customers and at the depot at least some of the time during their last shift, however, most drivers reported not being able to maintain distance at all times particularly when at the depot. Face masks and hand sanitiser were commonly used by drivers during their shift. While face masks offer varying levels of protection to the wearer they help to prevent transmission from an infected individual to others.^28^ The majority of drivers received some protective items from their employers, however, less than half of drivers felt that they provided effective protection.

By 7 December 2020, there had been 1 770 619 confirmed cases of COVID-19 in the UK, accounting for approximately 2.6% of the UK population.^11 29^ We found 3.0% of delivery drivers surveyed had tested positive for SARS-CoV-2 since the start of the pandemic, slightly higher than the national estimate, and over a sixth of delivery drivers reported having to self-isolate due to a suspected or confirmed case of SARS-CoV-2. A small proportion of delivery drivers reported working with symptoms of COVID-19 or while a member of their household had a confirmed or suspected SARS-CoV-2 infection. The majority of drivers reported continuing to work in this situation due to financial reasons, this may be associated with statutory sick pay being unavailable for most drivers. Lack of access to paid sick leave is one of the main risk factors for respiratory infectious disease-related presenteeism.^30^ Providing wider access to paid sick leave may, therefore, help to improve adherence to public health measures such as self-isolation. Delivery drivers were critical to the pandemic response, ensuring supplies were available and providing a service to clinically vulnerable individuals shielding at home, highlighting the importance of minimising exposure risk for workers and customers. Further consideration is needed on how key workers can be best supported and protected in future public health emergencies.

Participants self-reported how many face-to-face interactions they had with customers and at the depot on their last working shift as a delivery driver. For most participants, their last working shift was during the same week as completing the survey, but a small proportion (approximately 2.5%) were recalling from a shift over a month ago. There is some risk of uncertainty in recall particularly with the small proportion of participants recalling from a less recent shift. This study may suffer from recruitment bias, the survey was conducted online only without a strict recruitment process. Mean number of contacts were calculated per shift and therefore cannot be directly compared with other contact studies which calculate the number of contacts per hour or per day. Our definition of a contact is an adaptation of the definitions used by other social contact studies, rather than using a definition set by an international public health agency, such as WHO.^14 25 31–33^ To reduce participant burden when reporting a large number of contacts, we kept the definition of a contact relatively simple. One motivation of this study was to quantify behaviours thought to be associated with transmission risk of SARS-CoV-2 to inform modelling studies, we hence used a similar definition to other pandemic contact studies to allow for comparisons across studies.^14 33^

Questions pertaining to SARS-CoV-2 infection and self-isolation referred to the time period from the start of the pandemic to completing the survey. Participants reported on their use of specific PPE items, however, we did not ask for any further details such as type of face mask worn or duration of use. Vaccination status of participants was not collected due to the timing of the study, which was released 5 days after the initial roll-out of the COVID-19 vaccine in the UK. As the vaccine was only available to clinically vulnerable individuals at this time, it was unlikely to be offered to participants during the study period.^34^ Delivery driver occupation was self-reported and not confirmed. However, this occupation was reported at the time of recruitment and before beginning the survey. We did not collect data on how long participants had been working as delivery drivers, therefore, estimations of the prevalence of SARS-CoV-2 infection and self-isolation among participants may not be an accurate representation of all delivery drivers.

## Data Availability

Data are available in a public, open access repository. The survey data are available from Lancaster University’s research directory at: https://doi.org/10.17635/lancaster/researchdata/553. License: Creative Commons Attribution licence (CC BY). The code to reproduce the statistical analyses is available at: https://doi.org/10.5281/zenodo.7517541.
