# Supplementary material for: Contact patterns of UK home delivery drivers and their use of protective measures during the COVID-19 pandemic: a cross-sectional study
Source: Occup Environ Med. 2023 Apr 13;80(6):333–8. doi: 10.1136/oemed-2022-108646 (PMC10314008; doi:10.1136/oemed-2022-108646)
Supplement: Supplementary data [file oemed-2022-108646supp001.pdf]

Supplementary Material - Contact patterns of UK home delivery drivers and their use of protective measures during the COVID-19 pandemic: a cross-sectional study

Jessica RE Bridgen; Centre for Health Informatics, Computing and Statistics, Lancaster Medical School, Lancaster University, Lancaster, UK
Hua Wei; School of Health Sciences Centre, University of Manchester, Manchester, UK
Carl Whitfield; Immunity and Respiratory Medicine, University of Manchester, Manchester, UK
Yang Han; Department of Mathematics, University of Manchester, Manchester, UK
Ian Hall; Department of Mathematics, University of Manchester, Manchester, UK
Chris P Jewell; Department of Mathematics and Statistics, Lancaster University, Lancaster, UK
Martie JA van Tongeren; Academic Health Sciences Centre, University of Manchester, Manchester, UK
Jonathan M Read; Centre for Health Informatics, Computing and Statistics, Lancaster Medical School, Lancaster University, Lancaster, UK
jonathan.read@lancaster.ac.uk (corresponding author)

Contents

Tables **2**

Survey questions.. **5**

Tables

Table S1: Participants' employment information. N is the number of participants who provided a response to the question.

	Number of participants (%)
Current employment situation (N = 162)	
Self-employed and completely independent	88 (54.3%)
Self-employed but receiving certain workers benefits	14 (8.6%)
Employed by one company - full time	46 (28.4%)
Employed by one company - part time	11 (6.8%)
Employed by multiple companies	3 (1.9%)
No response	0 (0.0%)
Current weekly working hours (N = 162)† *	
0	0 (0.0%)
1 - 10	6 (3.7%)
11 - 20	17 (10.5%)
21 - 30	17 (10.5%)
31 - 40	44 (27.2%)
41 - 50	42 (25.9%)
51 or more	36 (22.2%)
In receipt of statutory sick leave pay (N = 151)†*	
Yes	38 (25.2%)
No	103 (68.2%)
Not sure	10 (6.6%)

† Question required a response from the participant.
* As a delivery driver

Table S2: Adjusted incidence rate ratios for number of customer contacts per shift by select variables.

	Multivariable analysis	
	**Adjusted Incidence Rate Ratio (95%CI)**	**p-value**
Age		
18-29	1.65 (1.07-2.60)	0.020
30-39	1.13 (0.77-1.68)	0.525
40-49	1.64 (1.15-2.34)	0.004
50-59	1.00	-
60-69	1.00 (0.60-1.75	0.986
Sex		
Female	1.00	-
Male	0.98 (0.70-1.36)	0.910
Employment type		
Self-employed and independent	1.00	-
Self-employed and receiving some benefits	1.19 (0.74-1.97)	0.456
Employed by one company - full time	0.66 (0.47-0.94)	0.019
Employed by one company - part time	0.77 (0.39-1.58)	0.404
Employed by multiple companies	0.97 (0.40-2.80)	0.955
Furthest distance from depot to delivery (miles)		
0-9	1.00	-
10-19	1.18 (0.79-1.75)	0.422
20-99	1.23 (0.83-1.82)	0.274
100-499	1.07 (0.60-1.91)	0.805
Weekly working hours		
1-10	0.47 (0.24-1.00)	0.036
11-20	0.55(0.32-0.99)	0.018
21-30	0.44 (0.28-0.71)	0.001
31-40	1.00	-
41-50	0.95 (0.63-1.43)	0.806
51+	0.98 (0.65-1.48)	0.918
Items normally delivered		
Small parcels only	1.00	-
Large items only	1.24(0.55-3.13)	0.600
Small parcels and large items	0.88(0.59-1.34)	0.541
Groceries only	0.34(0.18-0.64)	0.001

Takeaway only	0.19(0.11-0.37)	<0.001
Other items only	0.43(0.24-0.80)	0.003
Other delivery type combinations	0.58(0.38-0.93)	0.014

Survey questions

Q1a. Are you aged 18 or over?

- ◯ Yes
- ◯ No

Q1b. Do you work as a home delivery driver in the UK? (including mail, parcels, homeware, takeaway, groceries etc.)

- ◯ Yes
- ◯ No

Q1c.
Please make sure you agree to the following before continuing with the survey:
You currently live in the UK;
You are a home delivery driver;
You have read the Participant Information Sheet and fully understand what is expected of you within this study;
Your participation is voluntary, and you are aware that you can stop the survey at any point; You consent to Lancaster University keeping the anonymised data for a period of 10-years after the study has finished;

- ◯ **I consent to taking part in the CoCoNet: Home Delivery Driver study**

Q2. Where in the UK do you currently live?

- ◯ England
- ◯ Northern Ireland
- ◯ Scotland
- ◯ Wales
- ◯ I do not live in the UK

Q3. What is your age?

- ◯ 18 - 29 years old
- ◯ 30 - 39 years old

- 40 - 49 years old
- 50 - 59 years old
- 60 - 69 years old
- 70 - 79 years old
- Aged 80 or over

Q4. What is your sex?

The answer you give can be different from what is on your birth certificate.

- Female
- Male
- Prefer not to say

Q5. Which of the following best describes your ethnicity?

- English / Welsh / Scottish / Northern Irish / British
- Irish
- Gypsy or Irish Traveller
- Any other White background
- White and Black Caribbean
- White and Black African
- White and Asian
- Any other Mixed / Multiple ethnic background
- Indian
- Pakistani

- Bangladeshi
- Chinese
- Any other Asian background
- African
- Caribbean
- Any other Black / African / Caribbean background
- Arab
- Any other ethnic group
- Prefer not to say

Q6. What is the first part of your home postcode?
For example, if your home postcode was LA1 4YW then you would enter LA1.

____

Q7. What is you highest level of education?

- Higher education and professional/vocational equivalents
- A levels, vocational level 3 and equivalents
- Trade apprenticeships
- GCSE/ O level grade A*-C, vocational level 2 and equivalents
- Qualifications at level 1 and below
- Other qualifications
- No qualifications
- Not sure

Q8. Are you self-isolating or shielding because of COVID-19?
A vulnerable individual here refers to a clinically extremely vulnerable person.

- ◯ I am not self-isolating or shielding
- ◯ Self Isolating - I have symptoms of COVID
- ◯ Self Isolating - Someone in my household has symptoms of COVID
- ◯ Self Isolating - Someone in my support bubble has symptoms of COVID
- ◯ Self Isolating - Someone I have been in contact with has symptoms of COVID
- ◯ Self Isolating - Told to do so by a contact tracer
- ◯ Self Isolating - Told to do so by a contact tracing phone app
- ◯ Self Isolating - In travel-related quarantine
- ◯ Shielding - I am a vulnerable individual
- ◯ Shielding - I live with a vulnerable individual
- ◯ Other - *please do not include any identifying information*

____

- ◯ Not sure

Q9. Have you ever had to self-isolate due to COVID-19 infection (either suspected or confirmed)?

- ◯ Yes
- ◯ No

Q10. In which month(s) have you had to self-isolate? Tick all that apply.

- ☐ March 2020
- ☐ April 2020
- ☐ May 2020
- ☐ June 2020
- ☐ July 2020
- ☐ August 2020
- ☐ September 2020
- ☐ October 2020
- ☐ November 2020
- ☐ December 2020

- ☐ January 2021
- ☐ February 2021
- ☐ March 2021

Q11. Have you ever tested positive for COVID-19?

- ◯ Yes
- ◯ No

Q12. In which month(s) did you test positive? Tick all that apply.

- ☐ March 2020
- ☐ April 2020
- ☐ May 2020
- ☐ June 2020
- ☐ July 2020
- ☐ August 2020
- ☐ September 2020
- ☐ October 2020
- ☐ November 2020
- ☐ December 2020
- ☐ January 2021
- ☐ February 2021
- ☐ March 2021

Q13. How many other people currently live with you at home?

- ◯ 0 - I live alone
- ◯ 1
- ◯ 2
- ◯ 3
- ◯ 4
- ◯ 5 or more

Q14. How many people of each age group live with you at home?
Do not include yourself.

Drop down options of 0, 1, 2, 3, 4, 5 or more for each age group.

0 - 9 year olds
10 - 19 year olds
20 - 29 year olds
30 - 39 year olds
40 - 49 year olds
50 - 59 year olds
60 -69 year olds
70 - 79 year olds
Aged 80 or over

Q15. What do you normally deliver?

- ▢ Letters and mail
- ▢ Small parcels
- ▢ Large items (e.g. large appliances, furniture)
- ▢ Takeaway food
- ▢ Groceries
- ▢ Other - please specify ____

Q16. What is your current employment situation?

◯ Self-employed and completely independent

◯ Self-employed but receiving certain workers benefits (e.g. holiday pay, sick pay). Please specify - do not include any identifying information

____

◯ Employed by one company - full time

◯ Employed by one company - part time

◯ Employed by multiple companies

Q17. How many hours do you currently work as a delivery driver per week?

◯ 0 hours

◯ 1 - 10 hours

◯ 11 - 20 hours

○ 21 - 30 hours

○ 31 - 40 hours

○ 41 - 50 hours

○ 51 hours or more

Q18. How many deliveries did you make during your last shift?
____

Q19. When was the last shift you worked as a delivery driver?

○ This week

○ Earlier this month

○ Last month

○ 2 months ago or more

Q20. Did you use your own vehicle to make deliveries on your last shift?

○ Yes

○ No

Q21. During your last shift, how many customers did you meet face-to-face?
____

Q22. On your last shift, which types of face-to-face contact did you make with customers? Tick all that apply.

- ☐ Brief face-to-face interaction with customer (less than 5 minutes)
- ☐ Prolonged face-to-face interaction with customer (more than 5 minutes)
- ☐ Entered a customer's property (e.g. for installation or to drop off a heavy item)
- ☐ Hand signature required from customer
- ☐ Other, please specify ______________________________________

Q23. Were you able to maintain social distance (at least 2 metres apart) from all of the customers you met on your last shift?

- Yes, all of the time
- More than half of the time
- Less than half of the time
- No, none of the time
- Not sure

Q24. During your last shift, how many people did you meet in the location where you collect items for delivery (e.g. depots or food outlets)?
Only include those you had a face-to-face conversation with.

____

Q25. Were you able to maintain social distance (at least 2 metres apart) from everyone you met in the location where you collect items for delivery last shift?

- Yes, all of the time
- More than half of the time (2)
- Less than half of the time
- No, none of the time
- Not sure

Q26. During your last working week, what was the furthest distance you travelled from a collection point (at a depot or restaurant) to a delivery address?

- 0 - 9 miles
- 10 - 19 miles
- 20 - 99 miles
- 100 - 499 miles
- 500 miles or more

Q27. During your last working week, did you share a vehicle with a colleague to make deliveries? (e.g. for large items)

◯ Yes

◯ No

Q28. During your last working week, did you always share a vehicle with the same colleague?

◯ Yes

◯ No

Q29. What measures have you taken to prevent infection when sharing a vehicle? Tick all that apply.

- ☐ Keeping windows open
- ☐ Wearing a facemask
- ☐ Using hand sanitiser
- ☐ Other, please specify _________________

Q30. On your last shift, did you use any Personal Protective Equipment (PPE)? Tick all that apply.

- ☐ Facemask
- ☐ Gloves
- ☐ Hand sanitiser
- ☐ Other, please specify _________________

Q31. Was the PPE provided by your employers or contracting companies/platforms?

◯ Yes

◯ No

◯ Not sure

Q32. Do you think they are effective protection?

◯ Yes

◯ No

◯ Not sure

Q33. Do you think you have adequate knowledge and information about workplace COVID-19 risk?

◯ Yes

◯ No

Q34. Where do you learn about workplace COVID-19 risk? Tick all that apply.

- ▢ Company/platform communications
- ▢ Government website
- ▢ News and social media
- ▢ Other, please specify ______________________

Q35. Do you receive statutory sick leave pay for your delivery occupation?

- ◯ Yes
- ◯ No
- ◯ Not sure

Q36. How would you cover your living expenses if you had to take time off work due to being ill with COVID-19 or COVID-19 symptoms? Tick all that apply.

- ▢ Special COVID-19 funding scheme provided by the contracting companies/platforms
- ▢ Government self-employment income support scheme
- ▢ Social benefits
- ▢ Other, please specify _____________________

Q37. Since March 2020, have you worked whilst being ill with COVID-19 symptoms, or with a member of your household having a suspected or confirmed case of COVID-19?
COVID-19 symptoms include: a high temperature, a new continuous cough, a loss or change to your sense of smell or taste

- ◯ Yes
- ◯ No
- ◯ Not sure

Q38. What was the main reason you still went to work? Tick all that apply.

- ▢ Financial reasons
- ▢ Unable to find someone to cover my shifts
- ▢ Asked to or encouraged to carry on working by my employers
- ▢ Other, please specify ___________________________________
